# Impact of Housing and Infrastructure on handwashing in Peru

**DOI:** 10.1093/inthealth/ihaa008

**Published:** 2020-04-01

**Authors:** Nipher M Malika, Guisella Barbagelatta, Mary Penny, Kelly A Reynolds, Ryan Sinclair

**Affiliations:** Loma Linda University School of Public Health, 11188 Anderson Street, Loma Linda, CA 92350, USA; Instituto de Investigacion Nutricional, Av. La Molina 1885 Peru; Instituto de Investigacion Nutricional, Av. La Molina 1885 Peru; University of Arizona, Mel and Enid Zuckerman College of Public Health, 1295 N. Martin Avenue Tucson, AZ, USA; Loma Linda University School of Public Health, 11188 Anderson Street, Loma Linda, CA 92350, USA

**Keywords:** handwashing, living conditions, hygiene practices, slums, Peru

## Abstract

**Background:**

The metropolitan area of Lima, Peru has a third of the nation’s population living in slum dwellings that are hypothesized to contribute to inefficient household hygienic practices. The purpose of this study was to quantitatively assess which living conditions have the greatest impact on handwashing practices.

**Methods:**

A cross-sectional epidemiological design of participants ≥16 y of age from San Juan de Miraflores, a slum on the outskirts of Lima, Peru, was used. Poisson regression was applied to assess the impact of living conditions on handwashing practices.

**Results:**

We could not demonstrate a relationship between living conditions (home structure, overcrowding, water, grey water disposal) and reported handwashing. The reported lack of handwashing is associated with the number of children in the home (those with children <5 y of age were more likely not to report washing their hands) and length of stay in the slum in years.

**Conclusions:**

Living conditions play an important role in one’s health, therefore improved study designs are needed to determine which strategies are likely to be the most effective in improving outcomes for slum dwellers.

## Introduction

Our world has been undergoing a radical ecological change, with growth in cities rising from 5% to 50% in the last couple of centuries.^1^ In many low- and middle-income countries, this urban growth has been accompanied by slum settlements in the cities and on the outskirts, creating the urbanization of poverty.^1,2^ A slum, as defined by the United Nations Human Settlement Programme, ‘is a group of individuals that live under the same roof that lack one or more of the following conditions: access to improved water, access to improved sanitation, sufficient living space, durability of housing and secure tenure’.^2^ The United Nations estimates that 32% of the world’s urban population lives in slums; a population that is expected to double by 2030.^2^ In Peru, slum dwellers make up a third of the nation’s population,^3^ living in substandard housing with environmental conditions hypothesized to promote inefficient hygienic practices.^4,5^

Improvements in social determinants of health such as economic stability, healthcare, living conditions and education generally have been shown to improve health.^6,7^ For instance, access to clean water has been shown to reduce bacterial infections and promote health and well-being,^8^ and epidemiological research in Peru has argued for a distribution of social and economic development to address health issues.^9^ However, because many of these factors have occurred at the same time (e.g. improved public safety, literacy and living conditions), the specific impact of any single intervention is difficult to measure. Additionally, such interventions are typically evaluated relative to urban or rural health,^10^ but less is known about the impact on slum health. This is particularly the case for living conditions in regard to personal hygiene and handwashing.

Slums are synonymous with poor living conditions and with health outcomes that are worse when compared to neighbouring urban or rural areas.^1^ Conversely, improved hand hygiene practices at the individual and community level have had a major role in reducing morbidity and mortality.^11,12^ Handwashing with soap is the most cost-effective intervention for many illness, can reduce diarrheal disease by 47% and has the potential to improve millions of lives.^13^ Several studies have highlighted that handwashing accompanied with basic hygiene behaviour (respiratory, food, laundry, personal and oral hygiene) can prevent cholera, norovirus, listeria, salmonella, diarrheagenic *Escherichia coli* and acute respiratory infection.^13–17^ But despite the extensive research on hygiene practices, poor living conditions and their association with health, there is limited information on the extent to which living conditions affect household hygiene practices such as handwashing or cleaning. Therefore this study aimed to assess the impact different living conditions have on hand hygiene in an urban slum settlement. The authors hypothesized that better housing and infrastructure will yield better handwashing practices and mothers will have better handwashing practices because their hygiene behaviour directly impacts the health of their children.

## Methods

### Study population

**Figure 1.**
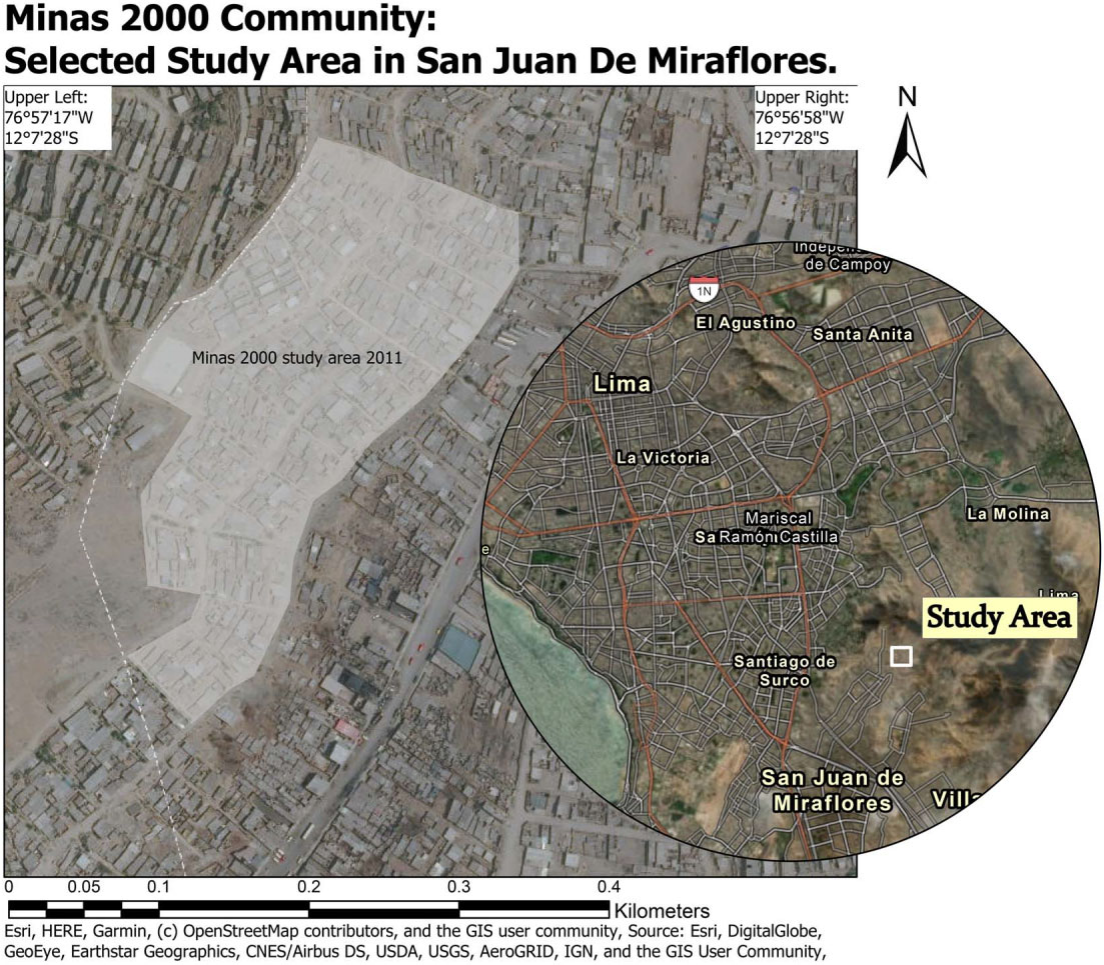
Map of San Juan De Miraflores

This study population consisted of 251 families living in the Minas 2000 area of San Juan de Miraflores, Lima, Peru; a young town populated by people who moved to the urban area in order be close to the urban centre of Peru. The Minas 2000 community is an informal settlement with community-defined boundaries that is located on the edge of the city of Lima in the San Juan de Miraflores District (Figure 1). In 2011, the Minas 2000 community was organized as a commune with a community kitchen that resident family households can use for meals.

Most residents live in homes made of a variety of material; some have electricity, but many lack running water or plumbing. Additionally, residents in San Juan de Miraflores use 10 times less water than families with running water but paid nearly 10–15 times more for it and were forced to store it in private cisterns, plastic barrels or garbage cans outside their homes.^17^ The area is a high risk due to frequent landslides as a result of soil instability, and homes are mostly built with unstable materials such as mats, plywood, drywall or cardboard, without technical criteria to evaluate the homes’ potential for low durability.

### Study design

Data collection occurred from August to November 2011. Approval from the Institute of Nutritional Research Institutional Review Board (IRB) for the protection of human subjects was obtained to perform the study. All 270 households within the Minas 2000 boundary were included in the assessment. The entire community was recruited for the study through their contacts with project facilitators employed at the Institute for Investigational Nutrition (IIN) located in the La Molina area of Lima, Peru. The Minas 2000 community’s commune chief ensured that all residents participated in the study, with 19 of the initial 270 not answering the primary outcome variable about handwashing with soap. Employees and consultant field staff of the IIN were trained with the required human ethics course (IRB) and how to administer the survey questionnaire. The Spanish-language questionnaire contained closed and open-ended questions that were pretested on a comparable population not participating in the study and later modified to improve questions found to be unclear. The topics covered in the questionnaire included hygiene and sanitation practices, water for drinking, disposal of used water, people living in the home and materials used to build the house. An additional observational study and a microbial surface sampling study took place while this survey questionnaire was administered.

Additional data were collected through in-home sampling (swabbing of frequently touched surfaces in latrines and kitchen areas) and direct observations that lasted for a typical 8-h work day.^18^ The results of the in-home sampling were published^18^ and indicated an increased risk for *Listeria monocytogenes* infection as the proportion of clean surfaces decreased.

The outcome variable in this study, handwashing with soap, was collected through a self-reported response to the question, ‘How many times per day did you wash your hands with soap and water or detergent?’ Two methods were used in this study to minimize bias in self-reported handwashing. The first method was written into the survey questionnaire process and asked the question using the strategy of activity recall to reduce overreported handwashing behaviour.^19^ To activate the memory of handwashing, the survey asked several related questions about hygiene practices in the home before asking for the frequency of handwashing with soap.

The second method to minimize self-report bias used an internal validation of the primary outcome variable. This internal validation relied on the substudy that was part of the larger parallel risk assessment^18^ published earlier. The substudy validated the handwashing variable by conducting 48 in-home observations among randomized households within the survey population. Each observation lasted 8 h in the home, and was undertaken by an investigator who was trained by the lead social scientist at the IIN to document hygiene-related observations. The observed and non-observed groups were measured for significance on the handwashing frequency variable. The study used a Mann–Whitney U test of independent means within SPSS Statistics for Windows, version 26 (IBM, Armonk, NY, USA) and did not find a significant difference (U=3857, p=0.323). This established that the self-reported frequency was not significantly higher than the mean rate among the observed households that had handwashing frequencies validated by trained personnel. The sample size and power assumptions for this internal validation were checked with G^*^Power 3.1^20^ a priori for the Wilcoxon–Mann–Whitney test, assuming an asymptotic relative efficiency method distribution. The actual power was calculated to be 0.99, with a type 1 error probability assumed to be 0.05 for the given subsample size of 48. This power was found to be sufficient to detect a difference in means using the above self-report validation method.

### Statistical analysis

The outcome variable (the number of times a person washed his/her hands with soap per event [using the restroom, cooking, eating, sneezing]) was created by summing reports of whether respondents washed their hands after defecating and urinating, before cooking, before eating and while sneezing in the midst of cooking. For instance, if a person used the restroom once in the 8-h recall period, began cooking, ate and sneezed over the course of a day, they should have washed their hands four times in that day. This variable was created from multiple synchronous variables to help minimize recall bias, as described above. This variable was then inversed for analysis and therefore predicted the number of times a person did not wash his/her hands.

A Poisson regression was used to analyse the relationship between handwashing and living conditions. Handwashing was adjusted for confounding factors such as age, education, income, parental status, number of children <5 y of age in the home and length of stay in the slum (in years), while measuring the impact of crowding within the household and water availability per day and home infrastructure (wall and floor makeup, the presence of a *hueco* in the front yard to dispose of grey water and if a bathroom was within the house). Some of these variables, overcrowding,^21,22^ water,^23,24^ children <5 y of age,^24^*hueco* (grey water disposal)^25^ and education,^26^ were associated with hygiene practice and health in previous studies. Crowding is related to the conditions of the dwelling as well as the space it provides—in this case it was measured in terms of the average living area per person in the place of residence.^27^

Two models were evaluated in the analysis. The first assessed if the amount of water each household had predicted handwashing, whereas the second model assessed if the living conditions themselves (type of house, location of bathroom, makeup of the bathroom) predicted handwashing.

## Results

Participants ranged from 16 to 69 y of age, with 51% having an education level less than high school and the majority (45%) of participants having a monthly income of 600–899 soles (US${\$}$183–273). Table 1 outlines the characteristics of the study population. There was an average of five people in each home, with a range of 2–14 people. Using the minimum acceptable living area per person, 44% of the participants lived in a crowded environment. How homes are designed, constructed and maintained (their physical characteristics) influences health and the ability of people to participate fully in hygiene-based practices such as handwashing.^28^ In this community, homes are built of materials such as cardboard (4.38%) and timber (86.85%), as well as sustainable materials such as brick (3.59%) and corrugated steel roofs (5.18%). The floors of the homes range from dirt (19.52%) and false floors (earthen floors; 22.71%) to cement (54.98%) or tile flooring (2.79%). The living conditions of participants, including kitchen/cooking arrangements and bathrooms, vary. There is no indoor plumbing and therefore each home purchases water. The amount of water each home has depends on the household income. Residents of San Juan paid 10–15 times more for water than those in Lima with running water. The storage containers are in some cases covered and at other times exposed to the elements, where mosquitoes can lay eggs. If possible, residents boil the trucked-in water before using it for cooking or drinking, but this adds to the financial strain they are already experiencing.

**Table 1.** Descriptive statistics for adults (n=251) in the Peru hygiene study

Characteristics	n (%)	Mean ± SD (interquartile range)
Age (y)		
≤19	7 (2.79)	18.42 ± 1.13 (16–19)
20–29	96 (38.25)	25.27 ± 2.85 (20–29)
30–39	113 (45.02)	34.23 ± 2.82 (30–39)
40–49	23 (9.16)	42.69 ± 2.43 (40–49)
≥50	12 (4.78)	56.83 ± 5.79 (50–69)
Parental status		
Mother	204 (81.60)	NA
Father	17 (6.80)	NA
Grandparent	15 (6.00)	NA
Other	14 (5.60)	NA
Education		
Less than high school	124 (51.39)	NA
High school graduate and above	120 (48.61)	NA
Income (soles)		
Do not know	18 (7.17)	NA
<200	6 (2.39)	NA
200–599	44 (17.53)	NA
600–899	113 (45.02)	NA
900–1499	53 (21.12)	NA
1500–2500	16 (6.37)	NA
>2500	1 (0.40)	NA
Children <5 y of age		
None	75 (29.88)	NA
1	147 (58.57)	NA
≥2	29 (11.55)	NA
Water (litres/day)		
0–100	130 (51.79)	50.5 ± 29.46 (0–85.71)
101–200	79 (31.47)	151.6 ± 34.77 (107.1–200)
≥201	42 (16.73)	326.1 ± 118.9 (214–642.85)
Crowding (persons per room in the home)		
Not crowded	139 (55.38)	1.15 ± 0.23 (0.66–1.5)
Crowded	112 (44.62)	2.56 ± 0.93 (1.67–6.0)
Wall material		
Cardboard, tin, mat	11 (4.38)	NA
Timber	218 (86.85)	NA
Seated brick	9 (3.59)	NA
Noble metal	13 (5.18)	NA
Floor material		
Dirt	49 (19.52)	NA
Cement (false floors)	57 (22.71)	NA
Cement (polished)	138 (54.98)	NA
Other better material	7 (2.79)	NA

The majority of participants, (51.79%) homes receive 0–100 litres of water per day, 31.47% received 101–200 litres and the remainder (16.73%) receive >201 litres but <643 litres per day. At a recommended rate of 50–100 litres of water per person per day needed to ensure that most basic needs are met and few health concerns arise,^27,29^ only 42% of the homes had a sufficient amount of water per day. The water received was used for cooking, cleaning, bathing and other household needs, but was not potable.

Mothers are hypothesized to have better handwashing practices because their hygiene behaviour directly impacts the health of their children.^30^ With this in mind, the handwashing practices of other adults were assessed in comparison with the mother. As outlined in Table 2, other adults in the home who were not a parent or a grandparent were more likely to wash their hands (RR 0.86 [CI 0.16 to 0.88]) when compared with the mother. Education and income were not significantly associated with handwashing. Those with one or more children <5 y of age were less likely to wash their hands. Additionally, although the RR indicates little difference, the duration of stay in the slum (in years) was also associated with the likelihood of not washing hands (RR 1.05 [CI 1.01 to 1.09]); longer tenure resulted in less handwashing after defecating and urinating, before cooking and eating and while sneezing in the midst of cooking. The amount of water each household received was not a significant predictor, nor was the number of people in the home.

**Table 2.** Model 1 results: does the amount of water predict hand hygiene?

Variable	β	RR	95% CI
Age	0.002	1.00	0.98 to 1.02
Education			
Less than high school	Ref	1.00	Ref
High school graduate and above	−0.09	0.92	0.70 to 1.17
Income	−0.13	0.88	0.78 to 0.98
Children <5 y of age			
None	Ref	1.00	Ref
1	0.55	1.74^**^	1.26 to 2.43
≥2	0.55	1.72^*^	1.06 to 2.74
Length of stay in the slum	0.04	1.05^*^	1.00 to 1.08
Parental status			
Mother	Ref	1.00	Ref
Father	0.35	1.42	0.87 to 2.20
Grandparent	0.02	1.02	0.50 to 1.94
Other	−0.86	0.42^*^	0.16 to 0.88
Crowding (in the home)			
Not crowded	Ref	1.00	Ref
Crowded	0.03	1.02	0.79 to 1.33
Water	−0.00	0.99	0.99 to 1.00

*p<0.05, **p<0.01, ***p<0.001.

In the second model, shown in Table 3, longer tenure in San Juan de Miraflores (RR 1.06 [CI 1.01 to 1.10]) and the number of children (one child <5 y of age: RR 2.00 [CI 1.43 to 2.85]; two or more children <5 y of age: RR 1.95 [CI 1.17 to 3.20]) in the home were associated with less handwashing. There was no significance, however, in variables that accounted for the infrastructure of the home, such as the wall makeup, floors, *hueco*, location of the restrooms or makeup of the restrooms.

**Table 3.** Model 2 results: do living conditions affect hand washing?

Variable	β	RR	95% CI
Age	0.002	1.00	0.98 to 1.01
Education			
Less than high school	Ref	1.00	Ref
High school graduate and above	−0.08	0.92	0.71 to 1.20
Income	−0.08	0.91	0.82 to 1.02
Children <5 y of age			
None	Ref	1.00	Ref
1	0.69	2.00^***^	1.43 to 2.85
≥2	0.67	1.95^**^	1.17 to 3.20
Length of stay in the slum	0.05	1.06^**^	1.01 to 1.10
*Hueco* (grey water storage/disposal)			
In the well	0.22	1.25	0.93 to 1.67
On the street	Ref	1.00	Ref
Other	0.24	1.27	0.83 to 1.89
Bathroom construction			
Silo space	−0.36	0.69	0.38 to 1.61
Wood-based cement silo	Ref	1.00	Ref
Granite silo based	−1.03	0.35	0.08 to 0.96
Silo with toilet	−0.14	0.86	0.63 to 1.18
Silo or ecological bathroom	0.06	1.06	0.60 to 1.78
Bathroom location			
Inside the house	Ref	1.00	Ref
Outside the house	0.12	1.12	0.77 to 1.59
Other	−0.49	0.61	0.14 to 1.69
Walls			
Cardboard, tin, mat	−0.32	0.72	0.33 to 1.40
Timber	Ref	1.00	Ref
Seated brick	−0.43	0.64	0.25 to 1.36
Noble metal	0.13	1.13	0.59 to 2.02
Floors			
Dirt	Ref	1.00	Ref
Cement (false floors)	0.06	1.06	0.70 to 1.62
Cement (polished)	−0.16	0.84	0.58 to 1.25
Other better material	0.11	1.11	0.47 to 2.29

*p<0.05, **p<0.01, ***p<0.001.

## Discussion

This study aimed to assess the impact different living conditions have on reported hand hygiene in an urban slum settlement and found that although some do have an impact, others do not. Slum dwellers spend most of their time in housing and environmental conditions that have a direct link to their health. Unhygienic practices affect quality of life and educational development and in many cases can result in diseases^24,31^ that are preventable. Previous research^32,33^ has shown that changes to one’s environment, whether in the home or at school, enables improved handwashing behaviour, and structural factors such as time, accessibility of water and high-quality facilities influence how likely it is that people wash their hands. Therefore, since a key motivating factor for adoption of handwashing is environmental conditions (such as house structure, access to water supply and excreta disposal sources),^32,34^ our hypothesis sought to assess if those with poor house structures or less access to water supply and excreta disposal sources would have poor hand hygiene practices. Previous studies in other populations^35,36^ have indicated that less water access is associated with lower handwashing rates. However, our results showed no significance in spite of the low levels of available water.

Although we tested for a relationship between the home infrastructure and handwashing, we could not demonstrate an association. Living in a house made of cardboard as opposed to brick showed similar hand hygiene behaviours. In this population, the house infrastructure had no bearing on one’s hygienic practices. As a factor of the built environment, building and housing structures have a major influence on health.^37^ With areas in the home serving as reservoirs for microbial colonization, hand hygiene is important, especially in slum environments.^38^ Our results revealed that although hand hygiene behaviour is a complex interaction of many factors, no one theory can reliably predict handwashing behaviour.^39^ Studying hand hygiene has to take into consideration the behavioural, cultural and social factors in the context of housing and infrastructure. Although a home’s structure might influence factors of health such as handwashing, understanding what motivates hand hygiene behaviour in a slum culture^38^ might be critical to understanding if housing and infrastructure truly play a role in handwashing. For instance, in Bangladesh there is a culturally acceptable time period during the day for personal hygiene,^40^ in a study among Koreans, cultural normative attributes were identified as factors that predict handwashing^41^ and in settings where people are internally displaced, the disruption of cultural norms can alter handwashing practices.^42^ Culture, to some extent, strongly influences attitudes regarding handwashing and is worth exploring. Additionally, we could not find the hypothesized associations with overcrowding, water, grey water disposal practices and education.

The handwashing associations that we did confirm are related to the number of children in the home and the length of stay in the slum. Those with one or more children <5 y of age were less likely to wash their hands. Past findings have indicated that with children <5 y of age in the home, the gap between knowledge and the practice of handwashing is more apparent.^43^ One’s length of stay in the slum (in years), although significant to handwashing, had little increased difference in risk. It has been found that people born within slums, in close proximity to slums or who have resided in slums for longer periods of time tend to remain in them or move to them due to social ties related to common culture, language and similar income-generating activities.^44^ Behavioural practices, such as handwashing, within such a context also happen to be uniform; and since poor handwashing among slum residents has been shown to be prevalent,^45,46^ based on our results we inferred that if one stays in the slum for an extended time, poor hand hygiene practices become habits that are difficult to break. Additionally, the longer one is immersed in an environment, the more prone one is to adopting the cultures and norms of that environment and the more one’s health is affected. For instance, in a study assessing two types of slums in Kenya—one where a quarter of the residents were born in the community and had resided there longer and the other where only 5% of the residents were born in the community and were more recent residents—it was found that demographic indicators such as length of stay affect economic and health outcomes.^47^ Without a clear understanding of the cultural contexts that influence individual and societal behaviours, health and hygienic practices are only going to be partially understood.^48^ In the literature, crowding has been reported to be an indirect measure of hand hygiene practices;^49,50^ however, in our study we were unable to find a link.

Compared with mothers, other adults in the home were found to have better handwashing habits. This could be influenced by the fact that handwashing appears to be an aspirational behaviour rather than a health concern and therefore the perception of handwashing among non-mothers might be different.^51^ Mothers with more younger children are also more likely to not wash their hands compared with mothers with older children,^52^ and that may be the reason why these mothers are less likely to wash their hands. Another reason could lie in the fact that, due to the scarcity of water, mothers use less water on themselves in order to preserve the little they have for their children and family. Additionally, although mothers may have intended to improve their handwashing behaviour, their physical environment, social relations or constructed attitude towards handwashing may prevent them from doing so.^51^ For instance, a perception of poor water quality may be why some wash their hands and others do not.

We believe this study provides an important background for additional research into the effects of living conditions on hygienic practices. Past studies have indicated that one crucial motivating factor for adoption of safe hygienic practices is living conditions. As population growth continues to be particularly rapid in urban areas of less developed regions, health burdens will continue to increase unless an intervention is put in place to affect all determinants of health. Understanding how hand hygiene and living conditions are correlated will allow for better built interventions to assist with the health burdens among slum dwellers.

The results of the current study should be interpreted in light of several limitations. First, we relied on self-reporting; it is possible that social desirability and recall bias might have influenced the responses of our participants. To rectify this, we relied on an observational substudy to validate the reported handwashing rate. This is one of many methods^19^ that can be used to help explain the overreporting of handwashing that commonly occurs in hygiene assessments. The data were also collected through an interview procedure, which may have added some interviewer bias into the study. Therefore, in light of such biases, further detailed investigation is needed.

There are many issues concerning all aspects of hand hygiene that remain unresolved. While hand hygiene practices are simple, compliance with handwashing is about human behaviour and discovering what influences such behaviours. Living conditions can influence hygienic practices and therefore studies on how living conditions affect hygiene practices within slum populations need to be conducted. Living conditions should also be assessed beyond the house infrastructure and neighbourhood, including security, safety, family and social relations, conditions in school and economic and material resources. A better understanding of such factors will provide gateways to understanding which strategies are likely to be the most effective in improving outcomes for slum dwellers.
